# Inhibitory Effects of the *Lactobacillus rhamnosus* GG Effector Protein HM0539 on Inflammatory Response Through the TLR4/MyD88/NF-кB Axis

**DOI:** 10.3389/fimmu.2020.551449

**Published:** 2020-10-05

**Authors:** Yubin Li, Shaojie Yang, Jingxian Lun, Jie Gao, Xuefeng Gao, Zelong Gong, Yu Wan, Xiaolong He, Hong Cao

**Affiliations:** Department of Microbiology, Guangdong Provincial Key Laboratory of Tropical Disease Research, School of Public Health, Southern Medical University, Guangzhou, China

**Keywords:** postbiotics, *Lactobacillus rhamnosus* GG, inflammatory response, colitis, TLR4/MyD88/NF-кB axis

## Abstract

Inflammatory bowel disease (IBD) is a chronic and relapsing intestinal inflammatory condition with no effective treatment. Probiotics have gained wide attention because of their outstanding advantages in intestinal health issues. In previous studies, a novel soluble protein, HM0539, which is derived from *Lactobacillus rhamnosus* GG (LGG), showed significant protective effects against murine colitis, but no clear precise mechanism for this effect was provided. In this study, we hypothesized that the protective function of HM0539 might be derived from its modulation of the TLR4/Myd88/NF-κB axis signaling pathway, which is a critical pathway widely involved in the modulation of inflammatory responses. To test this hypothesis, the underlying anti-inflammatory effects and associated mechanisms of HM0539 were determined both in lipopolysaccharide (LPS)-stimulated RAW 264.7 macrophages and in dextran sulfate sodium (DSS)-induced murine colitis. Our results showed that HM0539 inhibited the expression of cyclooxygenase-2 (COX-2) and the expression inducible nitric oxide synthase (iNOS) by down-regulating the activation of their respective promoter, and as a result this inhibited the production of prostaglandin E2 (PGE2) and nitric oxide (NO). Meanwhile, we demonstrated that HM0539 could ultimately modulate the activation of distal NF-κB by reducing the activation of TLR4 and suppressing the transduction of MyD88. However, even though the overexpression of TLR4 or MyD88 obviously reversed the effect of HM0539 on LPS-induced inflammation, HM0539 still retained some anti-inflammatory activity. Consistent with the *in vitro* findings, we found that HM0539 inhibited to a great extent the production of inflammatory mediators associated with the suppression of the TLR4/Myd88/NF-κB axis activation in colon tissue. In conclusion, HM0539 was shown to be a promising anti-inflammatory agent, at least in part through its down-regulation of the TLR4-MyD88 axis as well as of the downstream MyD88-dependent activated NF-κB signaling, and hence might be considered as a potential therapeutic option for IBD.

## Introduction

In recent years, owing to the rapid development of microbiome research related to the interaction of microbes with the host, administering microbes into patients as well as modulating human microbiota have become a focus of new therapeutic strategies (1, 2). Since the term “probiotics” was defined, they have been shown to act through cellular and molecular mechanisms involving antagonistic action on pathogens, and they have been shown to improve protective immunity, reduce inflammation induced by foreign antigens, and strengthen the mucosal barrier (2). The specific mechanisms involved nevertheless deserve further investigation. Probiotics have become a hot topic of research because of their beneficial functions in the treatment and prevention of intestinal diseases resulting from their maintenance of intestinal homeostasis. Evidence has been provided for probiotics inhibiting harmful bacteria from adhering to and invading intestinal mucosa, important since such adhesion and invasion play a key role in triggering the activation of immune response in IBD (3, 4), and subsequently enhancing the mucosal barrier and regulating the balance of intestinal flora, and restoring the function of its destruction (5, 6).

The efficacy of probiotics is thought to be strain-specific, with the different strains acting through different mechanisms in promoting host health (1). Receptors on the cell surfaces of various microbially derived components appear to play a vital role in the interaction with microbe-associated molecules and signals arising transduction, thereby regulating the expression levels of various cytokines and soluble inflammatory mediators (7). Systematic reviews have suggested that the interaction of microbe-associated molecular patterns (MAMPs) with toll-like receptors (TLRs) or other mucosal pattern recognition receptors likely contribute to probiotic-mediated improvement of IBD (8). Several studies have also indicated that TLR4 is the prime sensor of Gram-negative bacteria-derived LPS *in vitro*, and that TLR4-mediated signaling overexpression promotes inflammation and intestinal damage in mice with dextran sulfate sodium (DSS)-induced colitis (9), whereas TLR4-deficient (TLR4-knockout) mice are protected against this condition (10). Hence, TLR4 signaling might play a critical role in intestinal tract injury and repair processes.

Recently, it has been indicated that apical TLR4 stimulation in intestinal epithelial cells and mucosal immune cells leads to a high-intensity immune surveillance (7). However, the binding of microbe-associated molecules like LPS to receptors can activate antigen-presenting cells and trigger inflammatory transcriptional conduction factors, such as nuclear factor kappa B (NF-κB), thereby stimulating the transcription of inflammatory mediators (11, 12). Interestingly, the pro-inflammatory cytokines produced by macrophages, in turn, can directly trigger the NF-κB signaling transduction pathway through an auto-regulatory feedback loop mechanism to further amplify the inflammatory response and result in gut tissue destruction (13). The growing evidence for TLR4 as the main and probably only receptor for LPS is compelling. The mechanism underlying the beneficial effects of probiotics is believed to be associated with immunomodulation mediated by the TLR4 signaling pathway (14, 15).

Probiotics raise the attractive possibility that altering bowel flora could facilitate intestinal homeostasis in humans, but reservations remain about whether probiotics in IBD should represent biological response modifiers (16). The proposed health benefits of probiotics, including considering them as a conventional therapy for IBD, have undergone increasingly rigorous scientific scrutiny. However, there remains a lack of strict guidelines on the assessment of the safety and efficacy of probiotics, especially for their use in treating vulnerable neonates and immune-compromised individuals. Critically ill or preterm neonates with potentially impaired intestinal integrity are at higher risk of probiotic sepsis due to translocation, and even at increased risk of a cytokine storm induction (17–20). Hence, probiotics research is still in its early stages, and many more studies need to be conducted to confirm the stability, antibiotic resistance, and safety of probiotics when used to treat IBD (16).

Interestingly, more recent evidence indicated that the viability of probiotics is not deemed necessary to exert the protective functions, as not all clinical benefits or functionary mechanisms are directly based on living bacteria (21). There is increasing evidence for the beneficial effects of several different probiotics strains depending on soluble factors secreted from them, with these factors recently denoted as “postbiotics” (22–24). Regarding *Lactobacilli*, which is the most commonly referred to of the several reported strains of probiotics (25), note that beneficial effects of p40 and p75 purified from *Lactobacillus rhamnosus* GG culture supernatant (LCS) have been reported (26, 27). p40 and p75 protect epithelial cells against cytokine-induced apoptosis through activation of EGFR and its downstream target Akt (28, 29). Subsequent investigations have shown p40 to modulate the transactivation of EGFR and result in the up-regulation of mucin secretion in mice and human colon cancer cells (30).

Recently, we used LC-MS/MS to identify a novel soluble protein from LCS and provisionally named this protein HM0539. Interestingly, HM0539 was nearly the most abundant of the at least 58 proteins identified in the LCS. Gene expression analysis and amino acid sequence alignment of HM0539 revealed low sequence identities with p40 and p75; HM0539 is, therefore, considered to be a novel potential effector protein (31). We thus have constructed successfully the recombinant HM0539 plasmid and prepared highly purified recombinant protein for functional study. A subsequent study found HM0539 exhibiting intestinal barrier protective function characteristics, specifically with respect to promoting secretion of mucin and improving gut permeability. By exploring its therapeutic potential, specifically by introducing intestinal barrier associated models *in vivo*, we have provided evidence for the potential usefulness of HM0539 in preventing intestinal barrier dysfunction, bacteria translocation, and liver injury. In this context, considering the protective effect of HM0539, the mechanism by which HM0539 might be indirectly involved in immune regulation still remains unclear. Our previous studies constitute the cornerstone laying the foundation for further exploration of this subject.

Even though the precise composition of probiotics is still under investigation, only a few reports have focused on the underlying mechanisms of anti-inflammatory bioactivities. Thus, in this study, we investigated the anti-inflammatory properties and potential molecular mechanisms of the involvement of HM0539 in LPS-stimulated RAW264.7 macrophages and DSS-induced colitis in a murine model.

## Materials and Methods

### Cell Culture

Cells of the murine macrophage cell line RAW264.7 were purchased from the American Center for Type Culture Collection (ATCC, Rockville, MD, USA). The cells were cultured in Dulbecco’s modified Eagle’s medium (DMEM) supplemented with 10% heat-inactivated fetal bovine serum (FBS, PAN Biotech, Aidenbach, Germany), 100 units/mL penicillin (HyClone, USA), and 100 mg/mL streptomycin (HyClone, USA) at 37°C in an incubator with 5% CO_2_.

### Analysis of Cell Viability

Cell viability was assessed by performing the methylthiazol tetrazolium (MTT) assay (32). RAW264.7 cells were inoculated into a 96-well plate at a density of 1×10^5^ cells/mL and incubated with 5% CO_2_ at 37°C. Afterwards, the cells were pretreated with specific concentrations of HM0539 (6.25, 12.5, 25, 50, 100 ng/mL) for 24 h, and subsequently stimulated with or without LPS (1 µg/mL, Sigma-Aldrich) for a further 24 h. Then, a volume of 20 μL of an MTT solution (0.5 mg/mL, Jiancheng, Nanjing, China) was added to each culture well. Each mixture was allowed to incubate for 4 h, and then a volume of 200 mL of DMSO was added to each one, and the plate was shaken at 320 g for 30 min. The absorbance was measured at a wavelength of 570 nm with a microplate reader (Tecan M200 PRO NanoQuant).

### Determination of NO Production

The production of NO in the culture supernatant was quantified by evaluating the nitrite content according to the standard Griess reaction (33, 34). RAW264.7 cells were inoculated at a density of 1×10^5^ cells/mL in a 96-well plate and incubated with 5% CO_2_ at 37°C. Cells were pretreated with dexamethasone (MCE, New Jersey, USA) or specific concentrations of HM0539 (25, 50, 100 ng/mL) for 24 h and then further stimulated with or without LPS (1 µg/mL) for 24 h. The cell culture medium (100 μL) in each case was collected and incubated with 100 μL of Griess reagent (Solarbio, Beijing, China) at room temperature for 15 min. At the end point of the treatment, the absorbance was measured at a wavelength of 550 nm in a microplate reader. NO (μM) levels were calculated on the basis of the sodium nitrite standard (35).

### Determination of the Production of PGE2 and Pro-Inflammatory Cytokines

RAW264.7 cells were seeded at a density of 1×10^5^ cells/well in 96-well plates and incubated with 5% CO_2_ at 37°C. The cells were then incubated with specific concentrations of HM0539 for 24 h, and further treated with or without LPS (1 µg/mL) for 24 h. Levels of secreted TNF-α, IL-1β, IL-6, IL-18, and PGE2 (36, 37) in the culture supernatant were measured by using an ELISA kit (Proteintech Group, Chicago, USA) following the manufacturer’s instructions, and the absorbance in each case was measured at a wavelength of 450 nm in a microplate reader.

### Transfection of TLR4 and MyD88 Genes in Cells

TLR4 and MyD88 were overexpressed in RAW264.7 cells respectively (38, 39). In brief, when the RAW264.7 cells reached 60% confluence, they were transfected with pcDNA3-TLR4-YFP plasmid DNA (Addgene, Cambridge, MA, USA) and pcDNA3-Myd88-CFP plasmid DNA (Addgene, Cambridge, MA, USA) using Lipofectamine 3000 Reagent (Invitrogen Life Technologies, CA, USA) following the manufacturer’s instructions. Twenty-four hours after the last transfection, the cells were starved for 24 h in totally serum-free culture before being stimulated with LPS (1 µg/mL) in the presence or absence of HM0539 for an additional 24 h.

### Western Blotting Analysis

The total protein was collected using trypsin with 0.25% ethylenediaminetetraacetic acid, PBS, and cell lysis buffer, and its quantity was measured by performing the bicinchoninic acid protein assay (40). The lysate (40 µg) was separated using SDS-PAGE and transferred onto polyvinylidene difluoride (PVDF) membranes (0.45 μm, Millipore, USA) by using a Trans-Blot TurboTM (Bio-Rad, USA). The membranes were blocked with 5% (w/v) bovine serum albumin (BSA) dissolved in Tris-buffered saline Triton-X100 (TBST), and each was incubated with a different primary polyclonal antibody, i.e., against TLR4 (1:1000), MyD88 (1:1000), TRAF6 (1:1000), IRAK1 (1:1000), IRAK4 (1:1000), NF-κB p65 (1:1000), p-NF-κB p65 (1:1000), IκB-α (1:1000), p-IκB-α (1:1000), COX-2 (1:1000), iNOS (1:1000), or GAPDH (1:20000), overnight at 4°C, and all dilutions were in TBST blocked with 5% BSA. The membranes were analyzed using ECL reagents.

### Encapsulation of HM0539

Zein-pectin (core/shell) nanoparticles loaded with HM0539 were prepared based on previously published methods (41, 42), but with appropriate modifications and improvements. A pectin solution (5% w/v) was completely dissolved in distilled water. The resulting solution was subjected to centrifugation, and soluble protein HM0539 (6% w/v) was added into the resulting supernatant. A zein solution (1% w/v) was completely dissolved in an 85% ethanol solution with CaCl_2_ (0.5% w/v). Afterward, the pectin-protein solution was added drop-wise into the zein solution through a sterile silicone tube (diameter, 0.8 mm) connected to a 23G syringe pump. Microspheres formed immediately after the pectin drops (50 μL/drop) came into contact with the zein solution. The resulting mixture was stirred continuously for 2 min to keep the microspheres from sticking to each other. The zein-pectin (core/shell) nanoparticles loaded with HM0539 (5 μg/bead) were washed with distilled water, naturally dried at room temperature, and then preserved at 4°C. As a negative control, zein-pectin core/shell nanoparticles without HM0539 were prepared (43).

### DSS-Induced Murine Colitis *In Vivo*

Adult female C57BL mice (6–8 weeks old, weighing 18–20 g) were obtained from the laboratory animal center of Southern Medical University (Guangzhou, China). To analyze the efficacy of the encapsulated HM0539 at preventing and treating inflammatory lesions of acute colitis, after one week of acclimatization the mice were divided into four groups (control group, DSS-induced colitis group, DSS-induced colitis with encapsulated HM0539 group, and encapsulated HM0539 group), with 10 mice per group (10, 44, 45). Experimental acute colitis was induced in mice by feeding them 3% DSS (molecular weight, 40 kDa) and allowing them to drink freely for 7 days (46). The mice were gavaged with or without HM0539 beads (10 µg/mouse/day), and simultaneously treated with DSS, until sacrificed. All mice under standard laboratory conditions were housed five per cage at a temperature of 23 ± 2°C, a relative humidity of 45% to 65%, a light/dark cycle of 12 h, and fed with standard laboratory diet and water. All animal care and experimental procedures were strictly performed following the guidelines of the Medical Ethics Committee of Southern Medical University (Guangzhou, China) and conformed to the protocol on animal protection and welfare.

### Immunohistochemistry for MyD88 and NF-κB p65

Paraffin-embedded samples after dewaxing were treated with a rabbit anti-MyD88 polyclonal antibody or a rabbit anti-NF-κB p65 polyclonal antibody (Proteintech Group, Chicago, USA) followed by being incubated with HRP-conjugated anti-rabbit (Proteintech Group, Chicago, USA) secondary antibodies. The staining index was calculated based on the proportion of positively stained cells and the intensity of staining (47).

### Statistics

Data are presented as mean ± standard deviation (SD). Statistical comparisons were performed using a one-way analysis of variance (ANOVA) test with SPSS software (20.0). A threshold of p < 0.05 was considered to be statistically significant.

## Results

### HM0539 Improved Cell Viability in LPS-Stimulated RAW264.7 Cells

**Figure 1:**
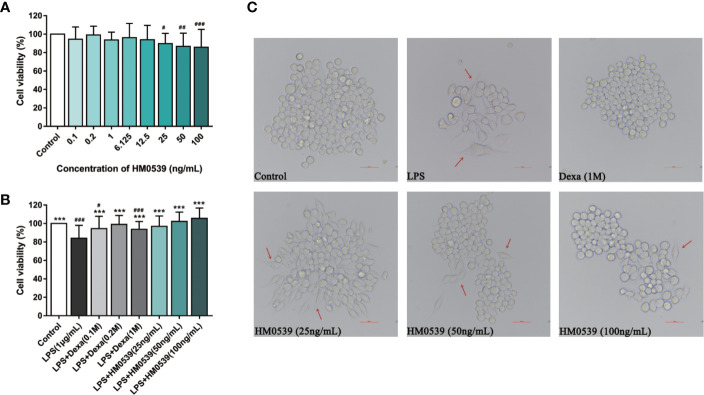
HM0539 improves cell viability in LPS-induced RAW264.7 cells. **(A)** The morphological changes in LPS-stimulated RAW 264.7 cells were viewed under a light microscope. The cells were incubated with the specific concentrations of HM0539 for 24 h **(B)** with LPS or **(C)** without LPS. Dexamethasone (0.1, 0.2, 1M) was used as a positive control, and cell viability was measured using the MTT assay. Red arrows indicate the differentiation and senescence of cells. The viability of cells for HM0539 at specific concentrations is expressed as a respective percentage of the non-treated control. Statistical analysis was carried out by performing a one-way analysis of variance test. Data shown are representative of at least three independent experiments, and indicate the mean ± SD (n=3). ^#^*p* < 0.05, ^##^*p* < 0.01, ^###^*p* < 0.001 compared with the control group, and ^***^p < 0.001 compared with the LPS-induced group.

LPS activated the inflammatory pathway in an auto-regulatory feedback loop through cell surface pattern recognition receptors and regulated inflammatory responses. In the absence of the positive control (dexamethasone), the viability of RAW264.7 cells was not significantly altered as a result of being treated for 24 h with HM0539 of several concentrations (**Figure 1A**). In a comparison of these results for cells stimulated by LPS (1 μg/mL) (**Figure 1B**), these results indicated that treatment of HM0539 significantly improved cell viability. Furthermore, cell morphology was examined using an inverted microscope. RAW 264.7 Cells cultured with HM0539 inhibited the differentiation and senescence of RAW 264.7 cells to some degree in morphological change (**Figure 1C**). Considering the results, subsequent experiments were performed using HM0539 at concentrations of 25, 50, and 100 ng/mL, respectively.

### HM0539 Reduced TLR4/MyD88/NF-κB Axis Signaling *In Vitro*

**Figure 2:**
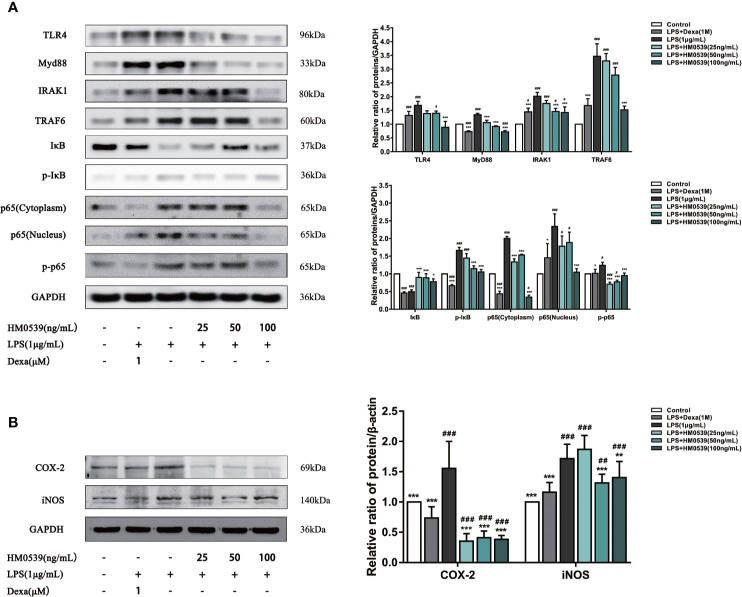
HM0539 attenuates inflammation *in vitro*. **(A)** Effect of HM0539 on the protein levels of TLR4, MyD88, IRAK1, TRAF6, IκB, p-IκB, and NF-κB p65 in RAW 264.7 cells. **(B)** Effect of HM0539 on the protein levels of COX-2 and iNOS. Dexamethasone was used as a positive control. The control level was normalized against its corresponding GAPDH, used as an internal control. The target protein was quantified using densitometry, and relative values of inhibition were calculated as the ratio relative to the control. Each experiment was performed five times independently and representative blots are shown. Statistical analysis was carried out by performing a one-way analysis of variance test. Data are presented as the means ± SD (n=5). ^#^*p* < 0.05, ^##^*p* < 0.01, ^###^*p* < 0.001 compared with the control group, and ^*^p < 0.05, ^**^p < 0.01, ^***^p < 0.001 compared with the LPS-induced group.

To further investigate the anti-inflammatory effect of HM0539 on LPS-induced inflammation, the related protein levels in the TLR4/MyD88/NF-κB axis were assessed in LPS-induced RAW264.7 cells. As shown in **Figure 2A**, the increase of TLR4 and MyD88 expression induced by LPS was suppressed by HM0539, and HM0539 decreased the phosphorylation of IκB in a dose-dependent manner. The phosphorylation and degradation of IκB might be involved in the initial step of NF-κB activation. Furthermore, HM0539 markedly inhibited the phosphorylation of NF-κB p65 and suppressed the translocation of NF-κB p65 subunits into the nucleus.

### HM0539 Reduced iNOS and COX-2 Protein Expression

The reduction in intracellular NO production is generally ascribed to the down-regulation of iNOS, a major pro-inflammatory enzyme that catalyzes the production of NO. COX-2, a rate-limiting enzyme that catalyzes the conversion of arachidonic acid to prostaglandins, is widely involved in inflammatory reactions. Compared with the control group, the levels of COX-2 and iNOS protein in the cells stimulated with LPS were significantly up-regulated. Importantly, as shown in **Figure 2B**, treatment of cells with HM0539 markedly suppressed the expressions of COX-2 and iNOS, and did so to an even greater extent than did the positive control. These results were consistent with the inhibitory effects of HM0539 on NO and PGE2 production described below.

### HM0539 Reduced Production of NO and Pro-Inflammatory Cytokines *In Vitro*

**Figure 3:**
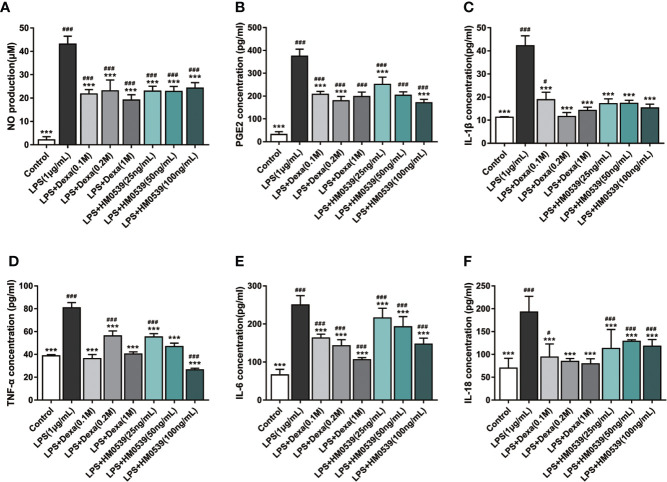
HM0539 attenuates inflammation *in vitro*. **(A)** Effect of HM0539 on NO production in LPS-stimulated RAW 264.7 cells. NO production was measured using the Griess reagent. **(B–F)** Effects of HM0539 on **(B)** PGE2, **(C)** IL-1β, **(D)** TNF-α, **(E)** IL-6, and **(F)** IL-18 production levels in LPS-stimulated RAW 264.7 cells. The relative inflammatory mediators were measured in the culture supernatants by performing an ELISA. Dexamethasone (0.1, 0.2, 1 M) was used as a positive control. Statistical analysis was carried out by performing a one-way analysis of variance test. Data are representative of at least three independent experiments and indicate the mean ± SD (n=3). ^#^*p* < 0.05, ^##^*p* < 0.01, ^###^*p* < 0.001 compared with the control group, and ^***^p < 0.001 compared with the LPS-induced group.

NO, as an indicator of NF-κB-mediated oxidative inflammatory response, facilitates the development of inflammatory diseases. As expected, the intracellular NO level increased markedly after the cells were stimulated with LPS, but decreased significantly after subsequent treatment with dexamethasone. Similarly, treatment of the cells with HM0539 was shown to significantly suppress LPS-induced intracellular NO production each of the several tested concentrations of before HM0539 was shown (**Figure 3A**). Besides, several pro-inflammatory cytokines could directly trigger the distal NF-κB signaling pathway, and thus their expressions were usually positively correlated with the activation of NF-κB signaling. The levels of pro-inflammatory cytokines expressed in the high-HM0539-dose group were somewhat, but not to a statistically significant level, lower than were those in the low-dose group. Thus, our results showed that exposure of LPS-stimulated macrophages to specific concentrations of HM0539 inhibited up-regulation of LPS-induced PGE2, IL-1β, TNF-α, IL-6, and IL-18 (**Figures 3B–F**).

### HM0539 Attenuated Inflammation Mediated by TLR4/MyD88/NF-κB Axis Signaling Pathway *In Vivo*

**Figure 4:**
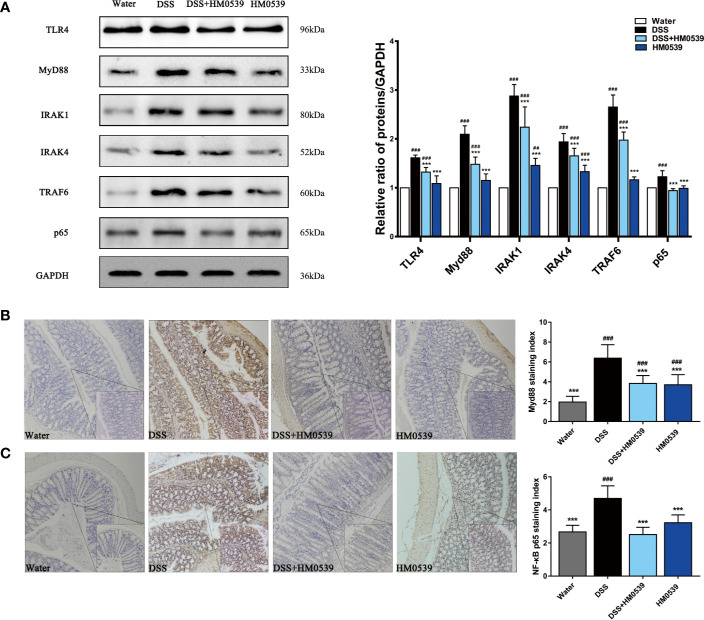
HM0539 attenuates inflammation *in vivo*. **(A)** Effect of HM0539 on the protein levels of TLR4, MyD88, IRAK1, IRAK4, TRAF6, and NF-κB p65 in mice. The control level was normalized against its corresponding GAPDH, used as an internal control. The relative intensity was calculated as the ratio of target protein to that of GAPDH and expressed as relative values of inhibition compared to the control group. **(B, C)** Effects of HM0539 on the levels of **(B)** TLR4 and **(C)** MyD88 based on immunohistochemistry assays (20×, 40× objective lens). For achieving statistical analyses of **(B)** MyD88 and **(C)** NF-κB staining indexes, each experiment was performed five times independently. Representative blots are shown. Statistical analysis was carried out by performing a one-way analysis of variance test. Data are presented as the means ± SD, with n=10 mice for each group. ^##^*p* < 0.01, ^###^*p* < 0.001 compared with the control group, and ^***^p < 0.001 compared with DSS-induced group.

Regarding the above *in vitro* investigations, the interaction between HM0539 and TLR4 in regulating signaling was preliminarily explored. Western blotting analysis further demonstrated that DSS significantly increased the expression of TLR4 protein compared with the control group. As shown in **Figure 4A**, DSS triggered significant increases in the levels of the TLR4, MyD88, IRAK1, IRAK4, and TRAF6 proteins of the TLR4/MyD88 signaling pathway and their downstream related proteins of NF-κB compared with the control group, while their expressions were to some extent yet significantly inhibited by HM0539, in a dose-dependent manner. Consistent with these results, immunofluorescence staining showed high densities of MyD88 (**Figure 4B**) and NF-κB p65 (**Figure 4C**) positive cells in the colon tissue of the DSS group, while expression decreased upon following the treatment with HM0539. Collectively, these results suggested that TLR4/MyD88/NF-κB signaling is involved in DSS-induced development of murine colitis, and that HM0539 plays a suppressive role in the DSS-induced inflammatory response.

### HM0539 Inhibited TLR4/MyD88/NF-κB Signaling Pathways

**Figure 5:**
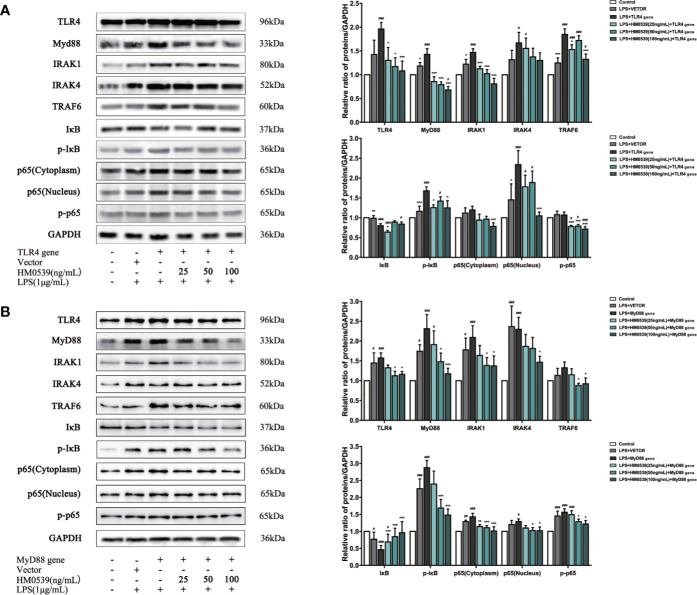
HM0539 inhibits NF-κB transduction associated with TLR4/MyD88-mediated signaling. **(A, B)** Effects of HM0539 on the expression levels of TLR4, MyD88, IRAK1, IRAK4, TRAF6, IκB, p-IκB, and NF-κB p65 after transfection of the **(A)** TLR4 gene and **(B)** MyD88 gene. Each experiment was performed five times independently and representative blots are shown. Statistical analysis was carried out by performing a one-way analysis of variance test. Data are presented as the means ± SD (n=5). ^#^*p* < 0.05, ^##^*p* < 0.01, ^###^*p* < 0.001 compared with the control group, and ^*^p < 0.05, ^***^p < 0.001 compared with the LPS + TLR4 gene or LPS + MyD88 gene group.

**Figure 6:**
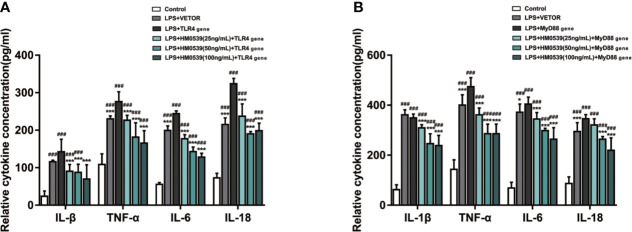
HM0539 inhibits NF-κB transduction associated with TLR4/MyD88-mediated signaling. **(A, B)** Effects of HM0539 on the levels of IL-1β, TNF-α, IL-6, and IL-18 in LPS-stimulated RAW 264.7 cells after transfection of the **(A)** TLR4 gene and **(B)** MyD88 gene. Each experiment was performed five times independently and representative blots are shown. Statistical analysis was carried out by performing a one-way analysis of variance test. Data are presented as the means ± SD (n=5). ^###^*p* < 0.001 compared with the control group, and ^*^p < 0.05, ^***^p < 0.001 compared with the LPS + TLR4 gene or LPS + MyD88 gene group.

To further investigate the mechanism by which HM0539 attenuated LPS-induced inflammation through the TLR4/MyD88/NF-κB axis signaling pathway, TLR4 was overexpressed in some RAW264.7 cells and MyD88 was overexpressed in other RAW264.7 cells. Our experiments showed that the expressions of proteins of the TLR4 signaling pathway and downstream related proteins (**Figures 5A, B**) and pro-inflammatory cytokines (IL-1β, TNF-α, IL-6, and IL-18 levels) (**Figures 6A, B**) in LPS-stimulated cells were significantly inhibited by HM0539, with this inhibition presented in a somewhat dose-dependent manner. Nevertheless, overexpression of transfected TLR4 or MyD88 dramatically reversed the effect of HM0539 while still maintaining its significant inhibitory effect. These results indicated that the MyD88-dependent pathway might be associated with an inhibition by HM0539 of the TLR4-mediated NF-κB hyperinflammatory signaling pathway.

## Discussion

IBD is a multifactorial immune disorder characterized by chronic inflammation and apoptosis of intestinal cells, which lead to intestinal mucosa damage, oxidative stress, and activation of immune cells with multiple inflammatory mediators (48–51). The soluble factors produced by probiotics, for which the term of “postbiotic” was recently coined, have been reported to alleviate the IBDs (22, 25, 52). Recently, we identified and purified a novel soluble protein from LCS, provisionally named HM0539, and it was found to protect the intestinal barrier (31). The results of our current study showed that HM0539 exerted protective effects strongly mediated by reducing the inflammatory response. Based on our observations of the effects of HM0539 in LPS-induced RAW 264.7 macrophages and DSS-induced murine colitis, we might expect HM0539 to serve as an agent of a prominent therapeutic strategy for treating IBD.

It has previously been reported that LCS ameliorated acute alcohol-induced development of intestinal and liver injury (53, 54). In addition, LGG has been observed to enhance the defense of a host intestine by regulating cell proliferation and apoptosis through a specific signal transduction system in the developing murine gut (55). But to the best of our knowledge, few studies have made a thorough inquiry into the precise composition of the active components and the underlying anti-inflammatory mechanisms of probiotics. Previous findings have shown both live and UV-inactivated LGG displaying similar effects on decreasing the inflammatory response, indicating that the anti-inflammatory effect of LGG may not depend on the bacteria being alive, but on the metabolites released by the bacteria during the fermentation process (21, 25, 56). As a consequence, scientific evidence for the beneficial effects of soluble LGG-derived factors is accumulating (25).

IBD refers to a heterogeneous group of diseases that present with an imbalance between inherent and acquired immunity as a result of its inevitably being accompanied by the production and release of different inflammatory cytokines in mucosal immune cells (57). Dysregulation of cytokines likely leads to the establishment of a characteristic inflammatory status in the intestinal tract. Furthermore, during the local inflammatory response in IBD, neutrophils and macrophages contribute to the recruitment of other immune cells, which leads to inflammatory cell infiltration (58). The gut, which is the largest independent immune system in the body, is integral to the recognition of various virulent pathogens and the immune protective antigen proteins (59). The gastrointestinal mucosa contains the largest pool of macrophages in the body. Macrophages are widely present throughout the gastrointestinal mucosa, especially distributed between the intestinal lamina propria and epithelial layer (60, 61). Macrophages act as a formidable regulator of host homeostasis, allowing the host to distinguish harmful antigens from foreign antigens and promoting recovery from chronic inflammatory and autoimmune diseases (7). Macrophages might be evaluated as potential targets for immune regulation of probiotics. In addition, we previously found HM0539 playing a role in the modulation of the immune response, as reflected by a resulting down-regulation of inflammatory cytokines including IL-1β, TNF-α, IL-6, and IL-18 produced by macrophages. Consequently, it is reasonable to think that macrophages are the main target cells of HM0539, which holds promise for investigating potential novel therapies to treat IBD.

It is clear that intestinal macrophages express a full range of TLRs, and even express most in humans, and intestinal macrophages are well established as a unique population of cells in maintaining mucosal homeostasis. Continuous exposure of intestinal mucosa to Gram-negative bacteria-derived LPS has been shown to activate a large number of immune cells and inflammatory cells, which usually leads to IBD or other severe diseases caused by microbial dysbiosis (62, 63). The mechanism of this process has been indicated to involve multiple upstream signaling pathways that subsequently augment the release of pro-inflammatory mediators. TLRs are widely distributed on the surfaces of epithelial cells and immune cells, and they function as pattern recognition receptors in the presence of LPS, thus establishing TLRs as receptors that lead to chronic inflammatory diseases (64, 65). Recently, several reports indicated that in the presence of organisms of the genus *Lactobacillus*, TLRs further serve as key initiators of innate immune responses and exert respective functions during homeostatic and inflammatory conditions (66, 67). Evidence was presented to demonstrate that *Lactobacillus jensenii* TL2937 is capable of attenuating the inflammation triggered by the activation of TLRs in porcine intestinal epithelial cells (68). In addition, *Lactobacilli* inhibited IL-8 production through inhibition of the TLR4 activation induced by *Helicobacter pylori* LPS (69). Furthermore, TLR4 was indicated to regulate the expression levels of inflammatory proteins and inflammatory cytokines in a model of LPS-induced murine sepsis. The pivotal signaling pathways involving TLR4 mediating inflammatory responses have been well established in various LPS-induced inflammatory diseases. In our current research, we speculated and then proved that the underlying anti-inflammatory effect of HM0539 was mainly accomplished by decreasing the levels of MyD88, TRAF6, p65 (nucleus and cytoplasm), and pp65 *via* reducing TLR4 expression. We overexpressed the TLR4 gene in RAW264.7 cells, and enhancement of TLR4 signaling in our experiments apparently reversed the effects of HM0539 on LPS-induced inflammation. TLR4 function was shown to involve differential engagement of MyD88-independent and MyD88-dependent signaling pathways—of note since these two pathways, used either simultaneously or one after the other, play a critical role in general in LPS-induced inflammatory response. Nevertheless, the former pathway has been shown to conduct the signal through more direct and simpler approaches than the latter. After the MyD88 gene was transfected into cells, the expression level of MyD88 and subsequent signal transduction were significantly enhanced, which also reversed the participation of HM0539 in the down-regulation of MyD88 and downstream signaling of NF-κB, but without a significant apparent effect on TLR4 expression. However, the treatment of HM0539, after overexpression of TLR4 or MyD88 by gene transfection, seemingly indicated its diminished role in signal regulation, but it still retained a certain degree of a suppressive effect. Therefore, the results of this study suggested that HM0539 might attenuate LPS-induced inflammation by interacting with the TLR4/MyD88/NF-κB axis and affecting its signal transduction.

Given that HM0539 has been shown to exert a potent beneficial effect on the integrity of the intestinal barrier (31), we hypothesized that it also might have a great potential ability to control the inflammation in mucosal immunity. In the present study, establishment of a model of DSS-induced experimental murine colitis was used to assess the anti-inflammatory potency of HM0539 *in vivo*. Although the mechanism underlying the role of DSS in inflammation has not been completely clarified, it is widely believed that the activation of macrophages is one of the mechanisms responsible for DSS-induced colitis in animals, which would echo the above-hypothesis mechanism derived from *in vitro* studies (70, 71). In addition, in our experiments, the developed encapsulation system apparently sufficiently protected the soluble protein HM0539 from the acid environment in the stomach and proteinase hydrolysis in the gastrointestinal tract when administrated orally. The zein-pectin (core/shell) nanoparticles, broadly used as a system for delivering drugs to the colon, were able to regulate drug release rates and improve the bioavailability (50). The results of western blotting and immunohistochemistry assays carried out *in vivo* were consistent with the results *in vitro*, which revealed an ultimate suppression by HM0539 of the activation of NF-κB and expression of downstream inflammatory mediators through its involvement in the modulation of MyD88-dependent pathways of the main TLR4 pathway.

Meanwhile, limitations of the present research are planned to be addressed in future research. We previously reported low similarities between the full-length amino acid sequence of HM0539 and the sequences of any of the TLR4 ligands, consistent with the idea that HM0539 cannot directly activate or bind to TLR4. Hence, the mechanisms of the interaction between HM0539 and TLR4 would be worth further thinking about and exploring. Interestingly, we did not find any significant effects of HM0539 on the expression of TLR4 in colonic epithelial cells isolated from mice, but promotion of mucin secretion and TJ protein expression and reduction in gut permeability were noted in this model. This result demonstrated an active involvement of HM0539 in the maintenance of intestinal tract homeostasis, which is not confined to TLR4-mediated modulation of innate immune response. On the other hand, we still could not rule out the possibility that HM0539 inhibits MAPK, Nrf2/HO-1, AP-1, NOD2, and NLRP3 signaling regulation of LPS-induced inflammatory response in the cells. Besides, numerous studies have indicated that TLR4-mediated inflammation conducts its signals mainly through two basic intracellular pathways, the MyD88-dependent and MyD88-independent pathways (72). In this process, LPS is considered to be the primary trigger, acting through both pathways (73). Activation of TLR4 through either the MyD88-dependent or MyD88-independent pathway has been shown to ultimately result in the priming of distal NF-κB-mediated pathways, with this priming taking considerably delayed to occur *via* the MyD88-independent pathway (74). Our study mainly focused on TLR4-mediated MyD88-dependent pathways, and we could not with certainty directly extrapolate from our studies whether the MyD88-independent pathway would be involved in the protective effect of HM0539. Therefore, further investigations are clearly needed to distinguish the roles of HM0539 in these two different parallel pathways. The potential for practical applications of this strategy still needs to be shown by carrying out further experiments with related gene-knockout animal models.

This study demonstrated that an HM0539-induced decrease of TLR4 expression might underlie the decreased MyD88 level, leading to the inhibition of distal NF-κB activation and pro-inflammatory mediators, thereby attenuating LPS-induced inflammatory responses. Further investigations of HM0539 on IBD still remain to be carried out and verified. It is possible to elucidate the anti-inflammatory effect of HM0539 at the molecular level, and to optimize and rationally use various probiotics components to achieve precise treatments. Based on previous and current studies, HM0539 deserves further consideration as a potential therapeutic agent for the treatment of IBD.

## Data Availability Statement

The raw data supporting the conclusions of this article will be made available by the authors, without undue reservation.

## Ethics Statement

The animal study was reviewed and approved by The Animal Care Committee of Southern Medical University.

## Author Contributions

HC, YL, SY, JL, XG, and YW conceived and designed the experiment. YL, SY, JL, XG, and YW performed the experiment. YL, SY, and JG analyzed the data. JL and XG contributed reagents/materials/analysis tools. YL, ZG, JG, YW, and XH participated in its design and coordination and helped to draft the manuscript. All authors contributed to the article and approved the submitted version.

## Funding

This project was financially supported by the National Science Foundation for Young Scientists of China (No. 81801985), China Postdoctoral Science Foundation (No. 2018M633076), and Undergraduate Training Program for Innovation and Entrepreneurship of Guangdong Provinces of China (No. 201612121071).

## Conflict of Interest

The authors declare that the research was conducted in the absence of any commercial or financial relationships that could be construed as a potential conflict of interest.
